# Standing up for Medical Microbiology

**DOI:** 10.1099/jmm.0.002036

**Published:** 2025-06-25

**Authors:** Lovleen Tina Joshi, Timothy J.J. Inglis

**Affiliations:** 1 Peninsula Dental School, Faculty of Health, University of Plymouth, Plymouth, UK; 2 Schools of Medicine and Biomedical Sciences, The University of Western Australia, Crawley, 6009, Australia; 3 PathWest Laboratory Medicine WA, QEII Medical Centre, Nedlands, 6009, Australia

**Keywords:** antimicrobial resistance, climate change, editors-in-chief, geopolitics, infection science, *Journal of Medical Microbiology*, open access

## Editorial

We are now at the point where the transition of the *Journal of Medical Microbiology* (*JMM*) Editor-in-Chief role switches from Tim Inglis to Tina Joshi while leading the journal to Open Access via the Subscribe to Open (S2O) model [1]. As part of our succession planning, we welcome our new Deputy Editor-in-Chief, Mathew Diggle, to the leadership team. The senior leadership group now spans three continents, reflecting *JMM*’s global reach and the international importance of medical microbiology [2].

This time of global turmoil and uncertainty within infection science and medical microbiology is challenging for biomedical journal leadership [3]. Geopolitical pressures, funding cuts and changing research priorities have led to a decline in the number of professionals working in the field, particularly in the USA [4,7]. In the United Kingdom, the higher education system is under immense strain as financial pressures have triggered many universities to implement widespread redundancies across a range of disciplines, including biosciences, medicine and dentistry [8,9]. Hence, we are losing our researchers and scientists from the field when their expertise is needed most. This presents significant challenges for the future workforce in maintaining expertise, knowledge and global collaboration at a time when antimicrobial resistance (AMR), climate change and new infectious diseases are emerging [10,11].

We believe that *JMM* has an important role to play in this context, as our journal helps to maintain the visibility, relevance and impact of medical microbiology research across the globe. Our international Editorial Board is vital to this effort and champions medical microbiology research voluntarily through handling submissions, reviewing and editing journal sections. The journal has transitioned to S2O, which is helping to broaden our reach, foster international engagement and support the advance of medical microbiology and infectious diseases at what is a critical time for the future of our discipline.

Despite the current climate, we have high ambitions for *JMM* and are committed to establishing it as the ‘Home of Medical Microbiology’ research for the community. We are building on the strong foundation established by our former co-Editors-in-Chief Norman Fry and Kalai Mathee to ensure *JMM* is resilient, robust and fit to withstand the volatile pressure of geopolitics. Indeed, *JMM* has much to look forward to. We have launched new article collections, such as the *Clostridioides difficile* *collection*, *Pathogen Genomics in Clinical Practice* and *Fungal Pathogenesis: Mechanisms, Resistance, and Threats*, and continue to welcome high-quality submissions in AMR, clinical microbiology and infectious diseases.

This would not be possible without the commitment of our Editorial Board, our reviewers, our readership, the Microbiology Society’s professional staff and authors who choose to submit their work to *JMM*. We want to remind our readers that the Microbiology Society is a not-for-profit publisher and that by publishing in *JMM*, they are helping to sustain the broader medical microbiology community. In times such as these, it is more important than ever to sustain our field. We can do this by supporting research, fostering collaborations and ensuring critical findings continue to reach a global audience. In this mission, *JMM* continues to stand up for medical microbiology.

## Abbreviations

AMR: antimicrobial resistance
*JMM*: *Journal of Medical Microbiology*
S2O: Subscribe to Open
